# The Biosynthesis and Medicinal Properties of Taraxerol

**DOI:** 10.3390/biomedicines10040807

**Published:** 2022-03-30

**Authors:** Ahmad Asnawi Mus, Lucky Poh Wah Goh, Hartinie Marbawi, Jualang Azlan Gansau

**Affiliations:** Faculty of Science and Natural Resources, Universiti Malaysia Sabah, Kota Kinabalu 88400, Malaysia; dx1811004t@student.ums.edu.my (A.A.M.); luckygoh@ums.edu.my (L.P.W.G.); hartinie@ums.edu.my (H.M.)

**Keywords:** taraxerol, in vitro, medicinal properties, biosynthesis, triterpenoids

## Abstract

Taraxerol is a pentacyclic triterpenoid that is actively produced by some higher plants as part of a defense mechanism. The biosynthesis of taraxerol in plants occurs through the mevalonate pathway in the cytosol, in which dimethylallyl diphosphate (DMAPP) and isopentyl pyrophosphate (IPP) are first produced, followed by squalene. Squalene is the primary precursor for the synthesis of triterpenoids, including taraxerol, β-amyrin, and lupeol, which are catalyzed by taraxerol synthase. Taraxerol has been extensively investigated for its medicinal and pharmacological properties, and various biotechnological approaches have been established to produce this compound using in vitro techniques. This review provides an in-depth summary of the hypothesized taraxerol biosynthetic pathway, the medicinal properties of taraxerol, and recent developments on tissue culture for the in vitro production of taraxerol.

## 1. Introduction

Despite recent advances in combinatorial chemistry, and other means of synthesis methods towards the production of essential drugs in healthcare, naturally derived compounds are still an invaluable source of medicine [1]. This is due to the minimal side effects and relatively higher biological activity of natural drugs compared to synthetic drugs [2]. Although the process of discovering effective drugs from natural raw materials is time consuming, costly, and less sustainable, advances in biotechnology could be helpful for such efforts [3]. In fact, natural products have played an important role in drug development whereby a considerable number of drugs are derived from naturally occurring compounds [4]. Today, there are more than 250 naturally derived drugs that are manufactured at large scales in the healthcare industries such as morphine, cephalosporin, and paclitaxel [5].

Taraxerol, an oleanane-type pentacyclic triterpene, is one of the natural compounds that have been investigated extensively for its potential utilization in drug development [6]. It has received major attention for its potential use as a therapeutic agent for the treatment of various diseases [7]. Plants containing taraxerol are *Hypericum perforatum* [8], *Clitoria ternatea* [9], *Mangifera indica* [10], and *Strobilanthes crispus* [11]. Taraxerol attracted wide interest among researchers due to its significant capabilities in modern pharmacology, such as its ability to act as an anti-tumor [12], anti-microbial [13], and anti-inflammatory agent [14], and in the treatment of Alzheimer’s disease [15].

Thus, this review aims to further explore the distribution of taraxerol in plants, their valuable properties and activities, as well as sustainable approaches in further producing this compound.

## 2. Taraxerol

**Figure 1:**
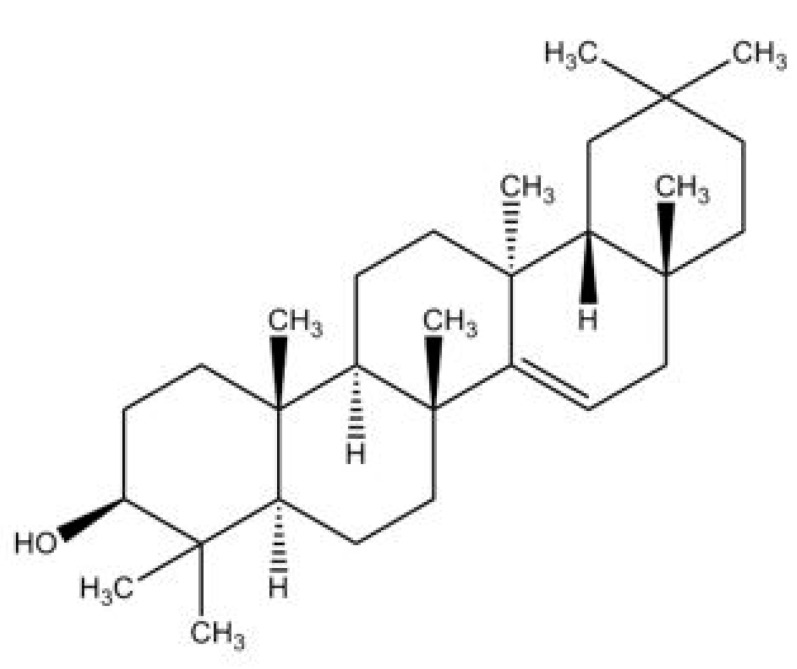
The proposed structure of taraxerol. Adapted from Beaton et al. (1955) [17].

Taraxerol, (3β)-D-Friedoolean-14-en-3-ol, is a pentacylic triterpenoid [6,16]. Its chemical structure was first elucidated by Beaton et al. (1955) who identified that the oleanane-3-ol lacks the methyl group at position 14, with an α-methyl substituent at position 13 and a double bond between positions 14 and 15 [17] (Figure 1). This compound is also known by a few other synonymous names, which are isoolean-14-en-3b-ol, skimmiol, alnulin, and tiliadin. Taraxerol can be extracted from various plant families and species found in nature. However, the synthesis of taraxerol is challenging and depends on natural resources that have a negative impact on biological conservation. Hence, the ongoing research on taraxerol production and its distribution provides vital information for future investigations.

### 2.1. Distribution of Taraxerol in the Plant Kingdom

Members of the Asteraceae family comprise the greatest number of taraxerol-containing taxa, followed by the Euphorbiaceae and Malvaceae families (Table 1). It should be noted that within the Euphorbiaceae family, species in the Euphorbia genus have shown considerable accumulation of taraxerol. The most prominent source of taraxerol was found to be chiefly concentrated in the leaves for most taxa [11,18,19,20,21,22,23,24,25,26,27,28,29,30,31,32,33,34,35,36,37], followed by the roots [9,12,38,39,40,41,42,43,44,45,46,47,48,49,50,51,52] and finally the stems [36,38,53,54,55,56,57,58,59]. Some literature has also managed to isolate taraxerol from flowers (Table 1). However, the distribution of taraxerol is highly diverse in plants, and taraxerol content differ in different parts of plants and across different plant species.

### 2.2. Biosynthesis Pathway of Taraxerol

**Figure 2:**
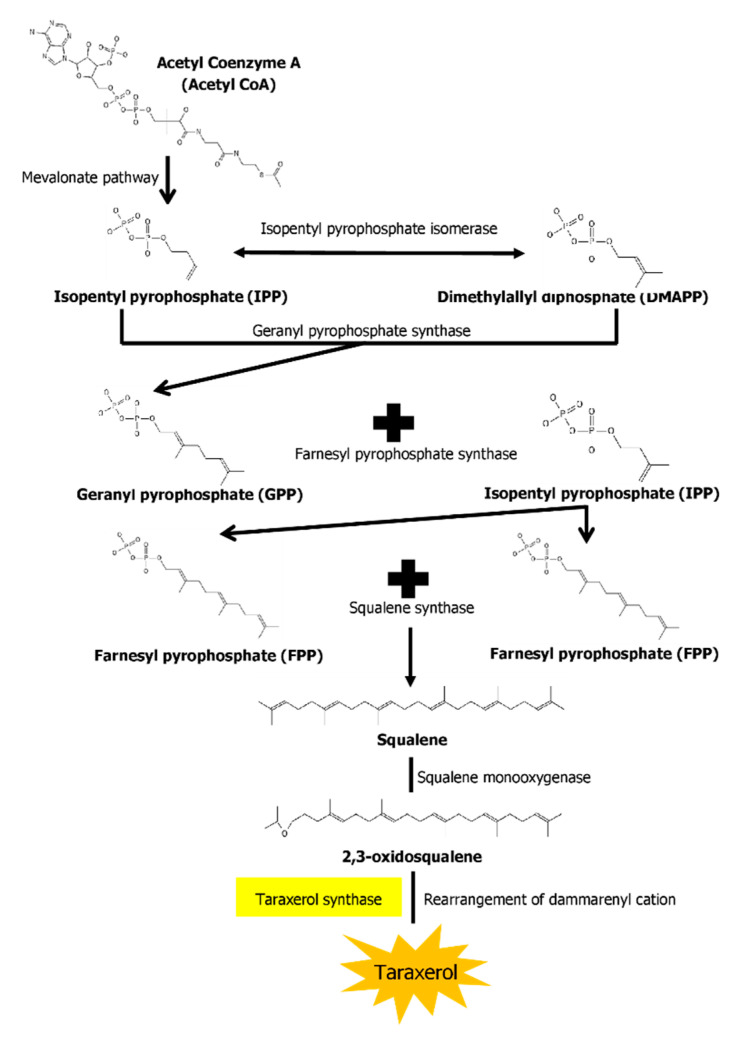
A summary of the biosynthesis pathway of taraxerol. With the aid of taraxerol synthase, dammarenyl cation undergoes rearrangements to produce taraxerol.

The biosynthesis pathways of taraxerol in plants have yet to be definitively elucidated. Swain et al. (2012) hypothesized that the biosynthesis of taraxerol in plants begins from the mevalonic acid pathway in the plant’s cell cytoplasm [94]. The mevalonate pathway begins with acetyl-CoA and ends with the production of IPP and DMAPP, which are the basic building blocks of various terpenoid compounds including taraxerol [95,96]. The DMAPP produced will then undergo condensation with IPP which is catalyzed by geranyl pyrophosphate synthase, producing geranyl pyrophosphate (GPP) that will be further subjected to condensation with IPP to produce farnesyl pyrophosphate (FPP) catalyzed by farnesyl diphosphate synthase (FPS) [29,97]. Squalene synthase catalyzes the condensation of the FPP molecules through reduction by NADPH to produce one molecule of squalene [98,99]. Squalene is then oxidised by NADPH and O_2_ to produce 2,3-oxidosqualene, which results in the reduction of NADPH into NADP^+^ and O_2_ to H_2_O [100]. 2,3-oxidosqualene is then utilised as a precursor for the biosynthesis of various triterpenoids, starting with a proton-initiated cyclization to produce dammarenyl cation, following which subsequent rearrangement leads to the pentacylic oleanyl cation via baccharenyl and lupenyl cation intermediates [101]. A series of 1,2-hyride shifts and/or methyl groups leads to compound rearrangements. Finally, the rearrangements of compounds via taraxerol synthase eventually lead to the formation of taraxerol in plants, more specifically in the cuticular waxes [72,100,102]. A summary of the biosynthesis pathway is illustrated in Figure 2.

## 3. Medicinal Properties of Taraxerol

### 3.1. Antioxidative Properties

‘Reactive oxygen species’ (ROS) is a term that encompasses various oxygen free radicals produced during cellular oxidative process. These compounds pose a significant risk factor for various diseases. Hence, antioxidants play an important role as a phytochemical that could inhibit the oxidative process. A study reported that taraxerol isolated from the bark of *Styrax japonica* exhibited weak radical-scavenging activity in the DPPH assay [103]. Increasing the concentration of taraxerol from 0.05–0.5 mg/mL yielded moderate radical scavenging activity in DPPH assay [85]. Jamila et al. (2015) supported the findings from Min et al. (2004), where taraxerol isolated from *Garcinia hombroniana* was found to be more potent than trolox and equipotent to gallic acid in DPPH radical scavenging activity, while in ABTS the scavenging activity of taraxerol was higher than trolox but less than gallic acid [71]. The reducing capacity of the extracts is related to the presence of biologically active compounds, particularly the hydrogendonating ability [17]. Owing to the potential chemical structure of taraxerol itself, this might explain the potent antioxidative capabilities of taraxerol. The current body of literature on taraxerol as an antioxidant provides valuable insight on this compound, but the work is not yet completed, and there are aspects that are under-explored.

### 3.2. Antimicrobial Properties

Singh et al. (2002) observed that 1 mg of taraxerol compound exhibited moderate antimicrobial activity against two Gram-positive (*Staphylococcus aureus* and *Bacillus thuringiensis*) and three Gram-negative bacteria (*Escherichia coli, Enterobacter cloacae*, and *Klebsiella pneumonia*) [13]. Koay et al. (2013) investigated the minimum inhibitory concentrations (MICs) of taraxerol on several bacteria and found that the compound is active against Gram-positive *Bacillus subtilis* and *Staphylococcus aureus* at a concentration of 15.6 µg/mL but is only moderately inhibitive to the Gram-negative *Escherichia coli*, *Klebseilla pneumonia*, and *Salmonella typhimurium* at a concentration of 62.5 µg/mL The taraxerol antimicrobial activity is comparable to that of positive control gentamicin [11]. Meanwhile, Hernandez-Chavez et al. (2012) reported on the anti-gardial activities of taraxerol towards *Giardia lambia*, a parasitic protozoan [104]. It was found that taraxerol possessed strong anti-gardial activity exhibiting a growth inhibition (IC_50_) of 50% at a concentration of 16.11 µg/mL and a growth inhibition of 90% at a concentration of 102.4 µg/mL, although the activity is lower compared to the positive control metronidazole. Another study on the cytotoxic activity of taraxerol against parasitic protozoans was conducted by Simelane et al. (2013), targeting malaria-causing *Plasmodium falcifarum* and *Plasmodium berghei* [105]. Anti-plasmodial activities were reported for taraxerol at a concentration of more than 100 µg/mL [105], but it was found to have no effect on mycobacteria (*Mycobacterium Madagascar* and *M. indicuspranii*), exhibiting a lower activity than the positive control chloroquine (IC_50_ = 14.1 ng/mL) [85]. Thus, future studies should focus mainly on the potential of taraxerol as an anti-protozoan drug. Warfield et al. (2014) conducted studies on the efficacy of taraxerol in combating the parasitic *Trypanosoma cruzi* [106]. The authors characterized the affinity of taraxerol with the sterol 14α-demethylase enzyme from *Trypanosoma cruzi* and found that the skeletal structure of taraxerol has higa affinity towards the enzyme, therefore providing potent inhibitory activity.

### 3.3. Anti-Fungal Properties

In an earlier study, taraxerol at a concentration of 1 mg/disc exhibited weak antifungal activities against four types of fungi namely *Aspergillus niger, Aspergillus flavus, Rhizoctonia phaseoli*, and *Penicillium chrysogenum* [13]. On the other hand, Aguilar-Guaddarama et al. (2009) shed some positive light on the anti-fungal potential of taraxerol [64]. The authors focused on another type of fungus known as dermatophytes, which are pathogens that cause skin diseases in animals and humans [107]. The compound exhibited strong anti-dermatophytic activities against various dermatophytes, at varying degrees of inhibition. Taraxerol was particularly effective against several species of *Trichophyton*, for instance *T. rubrum* and *T. mentagrophytes,* with an MIC of 12.5 µg/mL, as well as *Candida albicans* (MIC = 25 µg/mL) and *Aspergillus niger* at 100 µg/mL [64].

### 3.4. Cytotoxic Properties

Chaturvedula et al. (2004) found taraxerol at a concentration of 21.8 µg/mL was enough to inhibit 50% (IC_50_) of the growth of the A2780 ovarian carcinoma cell line, although it performed worse than the positive control doxorubicin (IC_50_ 1–3 ng/mL) [88]. At concentrations lower than 20 µg/mL, it showed little to no effect on the A2780 cell line [30]. Taraxerol also showed cytotoxicity towards the A431 squamous carcinoma cell line at 2.65 µg/mL, even though it was found to be inactive against HeLa, MCF-7, and MRC-5 cancer cell lines. While taraxerol cytotoxicity exhibited low activity compared to positive control doxorubicin, the activity is comparable to that of cisplatin [42]. Taraxerol also showed little to no inhibitory potential against Hypoxia-Induced Factor-1 (HIF-1) protein to reduce hypoxic tumor growth compared to 17-DMAF [17-(dimethylaminoethylamino)-17-demethoxygeldanamycin] [53]. However, taraxerol exhibited strong cytotoxicity towards human AGS gastric epithelial cell line at a concentration of 100 µmol/L by elevating cells arresting from complete mitosis and promoting early cell apoptosis rate from 4.45% to 10.29% [108]. Moreover, the report by Kaennakam et al. (2013) [49] contradicted earlier results from Csupor-Lötfer et al. (2011) [42] whereby the former observed that taraxerol displayed potent cytotoxicity to HeLa cells at a concentration of 14.94 µg/mL and to KB cells at a concentration of 13.58 µg/mL. Based on these results, taraxerol shows potential as a chemotherapeutic agent in cancer therapy.

### 3.5. Anti-Diabetic Properties

The utility of taraxerol in the treatment of diabetes was reported by Kwon et al. (2008), in which the compound was tested against the protein tyrosine phosphatase 1B (PTP1B)—a negative regulator of the insulin-signalling pathway for the treatment of type 2 diabetes [92]. Taraxerol was shown to exhibit moderate inhibitory properties against PTP1B at concentrations higher than 50 µM. Yet, Sangeetha et al. (2010) discovered that instead of targeting the PTP1B protein, taraxerol holds the potential to treat type 2 diabetes by dual action: as a glucose transport activator and as a glycogen synthesis stimulant [109]. The authors also revealed that taraxerol could reverse the effects of dexamethasone-induced insulin resistance back to its normal homoeostasis state. These findings were supported by Gururaja et al. (2015) who claimed that taraxerol is one of the active compounds that shows inhibitory activities against cholesterol esterase enzyme [18]. The antidiabetic properties of taraxerol were mostly attributed to its high affinity towards proteins involved in glucose metabolism [71].

### 3.6. Anti-Inflammatory Properties

Perhaps the most potent pharmacological properties actively shown by taraxerol is as an anti-inflammatory agent. Singh et al. (2002) investigated the anti-inflammatory activity of taraxerol on carrageenan-induced paw edema on rats and found that applying the triterpenoid extract at a dosage of 20 mg/kg led to edema reduction by 49.66% after 7 h [13]. Naik et al. (2004) further uncovered the anti-inflammatory effects of taraxerol on TPA-induced local inflammation in Swiss Albino mice, in which development of ear edema in rat model was suppressed following its application. A dosage of 1 mg/ear showed the best suppressive effects with a 25.7 mm difference in ear thickness 4 h following the injection [31]. Apart from paw and ear inflammation, taraxerol was also found to be beneficial in inflammatory pulmonary diseases. By directly acting on airway epithelial cells, taraxerol regulates the expression of the *Muc5a* gene in the cells, thus regulating mucus production in the inflamed airway [45].

Other than in the treatment of edema, taraxerol’s neuroinflammation amelioration effect has also been studied. Tsao et al. (2008) examined the effect of taraxerol on the production of nitric oxide (NO) and reactive oxygen species (ROS) by activated microglial cells, which play a number of deleterious roles in central nervous system mediation [24]. The NO and ROS are produced by activated microglial cells through the induction of NADPH oxidase (NOX) and nitric oxide synthase (NOS), which the authors noted to have been inhibited by 11.6% at 50 µM concentration and 50% at 24.2 µM concentration, respectively. The mechanism through which taraxerol functions as an anti-inflammatory agent was further elucidated by Yao et al. (2013) who showed that taraxerol downregulates the expression of proinflammatory mediators in macrophages through the interference of TAK1 and Akt protein activation, thus preventing NF-κB activation from producing various proinflammatory mediators through a cascade effect [110]. Cellular redox reactions have a critical role in the regulation of immune response, which directly suggested that taraxerol could also mediate inflammatory responses [110].

### 3.7. Treatment for Neurodegenerative Diseases

Taraxerol has also been extensively studied for its potential in treating neurodegenerative diseases. Cholinesterase enzymes were targeted by various target compounds in drug development to find possible treatments for neurodegenerative diseases, particularly Alzheimer’s [111]. Lee et al. (2004) found the potential of taraxerol for this purpose by inhibiting acetylcholinesterase (AChE) activity in a dose-dependent manner, with an IC_50_ value of 33.6 µg/mL [75]. This finding was supported by Jamila et al. (2014) in which taraxerol could not exercise its inhibitory effects at concentrations higher than 33.6 µg/mL [71]. Nevertheless, at 50 µg/mL, taraxerol exhibited inhibitory effects on butyrylcholinesterase (BChE) with 98.4% inhibition [71]. The IC_50_ of taraxerol against BChE was found to be at 17.8 µM.

Furthermore, taraxerol displayed high binding affinity to the monomers and mature fibrils of amyloid peptides, which are critical proteins associated with neurodegenerative disorders [111]. Taraxerol can completely assimilate into the human body and cross the blood-brain barrier, which are the two prerequisites for the development of a potent neurodegenerative drug [15]. In silico analysis of taraxerol affinity towards acetylcholinesterase A and B revealed high affinity towards both enzymes through the formation of hydrogen bonds [71]. This might explain the ability of taraxerol to compete for the active site of acetylcholinesterase, thereby exhibiting potential as a treatment for neurodegenerative diseases.

### 3.8. Other Notable Pharmacological Properties of Taraxerol

Taraxerol also exhibited wound healing properties. Naik et al. (2004) tested taraxerol for its inhibition on glycogen synthase kinase-3*β* (GSK-3*β*) protein, a wound healing biomarker through molecular and dynamic approach [31]. In silico studies have indicated that taraxerol may be a potent inhibitor of GSK-3*β* due to its expressed minimum binding (−12.59 kJ/mol) and docking energy (−11.25 kJ/mol). On the other hand, in vivo studies have shown that taraxerol displayed an astounding capability in healing three types of wounds, namely excised wounds (18.28 days with 94.42% enclosure), incised wounds (epidermal tensile strength of 562.36 g after 10 days of wounding), and dead space wounds (increased weight of granuloma tissues up to 21.02 mg, tissue breaking strength at 657.12 g, and hydroxyproline content of 1455.93 µg/100 g). Thus, the therapeutic properties of taraxerol can be extended to wound healing and remain to be further explored.

Natural compounds and extracts have been an important source for alternative medicine. The specific chemical compounds that have been isolated from natural plants hold a great potential in medicine, as had been demonstrated by the high number of FDA-approved drugs or natural products as well as their derivatives [112]. The search for antivirals is gaining popularity due to the coronavirus disease 2019 (COVID-19) which have had a huge impact on human well-being. Several phytochemicals such as friedelin, stigmasterol, and taraxerol were reported to exhibit promising antiviral properties [113]. Molecular dynamics simulation demonstrated that taraxerol has a better binding energy with viral proteins such as spike protein, main protease enzyme M^pro^, and the RNA-dependent RNA polymerase of COVID-19 [113]. This has shed light into further evaluation of taraxerol using in vitro and in vivo experiments for the development of a COVID-19 inhibitor.

## 4. In Vitro Production of Taraxerol

Cell culture techniques have emerged as an attractive alternative for the production of plants’ secondary metabolites, and various strategies have been developed for use in biomass accumulation as well as synthesis of a slew of secondary compounds [3]. However, taraxerol production through in vitro techniques has been limited so far. An example is the protocol developed by Swain et al. (2012) for producing taraxerol from *Clitoria ternatea* (Butterfly pea) through the establishment of transformed hairy root cultures [94]. Transformed hairy roots contained integrated *T_L_*-*rol*B gene and were able to increase taraxerol four-fold greater by dry weight basis compared to natural roots. Since transformation was involved in the process, the taraxerol isolated were ascertained by IR, ^1^H-NMR, and ^1^C-NMR spectroscopy as the modification of the *Clitoria ternatea* genetic make-up could change its phytochemical content.

Zafar & Sharma (2015) also used an in vitro approach to produce taraxerol through the establishment of callus cultures from the roots of *Taraxacum officinale* (Dandelion) [114]. Calluses were induced from the roots of *Taraxacum officinale* by using two types of MS media supplemented with 0.5 mg/L IAA + 1 mg/L BAP + 0.5 mg/L 2,4-D and 2 mg/L IAA + 1 mg/L BAP. The established root callus has successfully increased the taraxerol yield by 1.04 times. To further enhance taraxerol production, Zafar & Sharma (2015) established root callus suspension cultures using the same MS media and PGR combinations from Sharma and Zafar (2014) with the addition of methyl jasmonate (MJ) and *β*-cyclodextrin (CD) as elicitor agents [16,114]. According to the authors, both elicitors were able to elevate taraxerol production by 0.018% with MJ at 0.05 mM, 0.1 mM, and 0.2 mM, and by 0.023% with 25 mM *β*-CD compared to natural roots.

## 5. Conclusions

Taraxerol is a bioactive metabolite present in some higher plants which possesses multiple selective biological actions, especially in medicinal applications. Despite displaying little anti oxidative abilities and only moderate antimicrobial properties, various studies have reported the potential of taraxerol to act as an anti-plasmodial, antidiabetic, anticancer, anti-inflammatory, and anti-dermatophyte. These findings demonstrated the potential of taraxerol in the development of a novel and multipurpose drug. From a commercial point of view, taraxerol is hitherto a costly compound to chemically and biologically synthesize. With several pathways towards in vitro synthesis of taraxerol having already been established, it may not be a good use of resources to continue exploring more alternative synthesis pathways. Instead, research efforts should be directed towards optimizing known synthesis techniques through an experimental approach by the establishment of high-yielding cell lines, optimizing culture conditions, nutrient media, phytohormone contents and carbohydrate sources, elicitors, and precursors. With enhanced taraxerol production, further drug research and development works for various treatments using taraxerol can be performed.

## Figures and Tables

**Table 1:** The distribution of taraxerol isolated from different plant taxa.

Family	Genus	Species	Parts Extracted	Taraxerol Accumulation	Authors, [Ref.]
Acanthaceae	*Strobilanthes*	*S. callosus*	Aerial parts	0.69% for 5.0 Kg of plant material	[13]
		*S. crispus*	Leaves	N/A ^1^	[11]
Anacardiaceae	*Lannea*	*L. schimperi*	Stems, bark, and roots	299 mg/Kg dry weight	[38]
	*Mangifera*	*M. indica*	Leaves	0.4–0.9% yield ^2^	[18]
		*M. persiciformis*	Not specified	N/A	[60]
Annonaceae	*Uvaria*	*U. microcarpa*	Not specified	N/A	[61]
		*U. macrophylla*	Not specified	N/A	[62]
		*U. hookeri*	Bark of the roots	75 mg/Kg dry weight	[39]
		*U. narum*	Bark of the roots	0.04 mg/g dry weight	[39]
Apocynaceae	*Gomphocarpus*	*G. fruticosus*	Aerial parts	80 mg/Kg dry weight	[63]
Araliaceae	*Schefflera*	*S. octophylla*	Bark of the roots	N/A	[40]
Araliaceae	*Acanthopanax*	*A. trifoliatus*	Leaves	N/A	[19]
Asteraceae	*Artemisia*	*A. incisa*	Roots	36.67 mg/Kg dry weight	[41]
	*Conyza*	*C. canadensis*	Roots	4.27 mg/Kg dry weight	[42]
	*Ageratina*	*A. pichinchensis* var. *bustamenta*	Aerial parts	23.88 mg/Kg dry weight	[64]
	*Crossostephium*	*C. chinense*	Whole plants	N/A	[43]
	*Atractylodes*	*A. lancea*	Rhizome	N/A	[44]
	*Hieracum*	*H. pilosella*	Inflorescences	0.37% of 100 g of plant material	[65]
	*Taraxacum*	*T. officinale*	Roots	N/A	[12]
	*Chrysanthemum*	*C. morifolium* (I)	Flowers	0.2% yield ^2^	[66]
		*C. morifolium* (II)	Flowers	0.4% yield ^2^	[66]
	*Matricarcia*	*M. matricarioides*	Flowers	0.1% yield ^2^	[66]
	*Cosmos*	*C. bipinnatus*	Flowers	1.6% yield ^2^	[66]
	*Carthamus*	*C. tinctorius*	Flowers	0.6% yield ^2^	[66]
	*Taraxacum*	*T. platycarpum*	Flowers	0.5% yield ^2^	[66]
Betulaceae	*Alnus*	*A. nepalensis*	Leaves and twigs	19.7 mg (leaves) ^2^ 6 mg (twigs) ^2^	[20]
		*A. hirsuta*	Bark of the stems	3.03 mg/Kg dry weight	[53]
Braganiceae	*Cordia*	*C. multispicata*	Leaves	19.05 mg/Kg dry weight	[21]
Cactaceae	*Pereskia*	*P. aculeata*	Leaves	7.12% total abundance ^3^	[22]
	*Opuntia*	*O. dillenii*	Stems	N/A	[54]
Caesalpiniaceae	*Acrocarpus*	*A. faxinifolius*	Seed oils	N/A	[67]
Calophyllaceae	*Calophyllum*	*C. cordato-oblongum*	Twigs	N/A	[68]
Campanulaceae	*Adenophora*	*A. triphylla*	Roots	0.04 mg/g dry weight	[41]
	*Codonopsis*	*C. pilosula*	Not specified	N/A	[69]
		*C. pilosula* var. *volubilis*	Not specified	N/A	[70]
caryophyllales	*Pseudostellaria*	*P. heteraphylla*	Root tuber	N/A	[46]
Casuarinaceae	*Casuarina*	*C. equisetifolia*	Seed oils	N/A	[45,67]
Celastraceae	*Maytenus*	*M. undata*	Leaves	0.26 mg/g dry weight	[23]
Clusiaceae	*Garcinia*	*G. hombroniana*	Bark	2.31 mg/Kg dry weight	[71]
Crassulaceae	*Kalanchoe*	*K. daigremontiana*	Leaf	N/A	[72]
Ericaceae	*Vaccinium*	*V. iteophyllum*	Not specified	N/A	[73]
	*Rhododendron*	*R. ovatum*	Not specified	N/A	[74]
	*Vaccinium*	*V. oldhami*	Twigs	22 mg/Kg dry weight	[75]
	*Rhododendron*	*R. molle*	Roots	30 mg/Kg dry weight	[47]
Euphorbiaceae	*Sapium*	*S. baccatum*	Bark of the stems	3.25 mg/Kg dry weight	[55]
	*Euphorbia*	*E. hirta*	Stems	0.03 mg/g dry weight	[56]
	*Discocleidion*	*D. rufescens*	Bark of the roots	N/A	[48]
	*Thyrsanthera*	*T. suborbicularis*	Whole plant	13.67 mg/Kg dry weight	[76]
	*Euphorbia*	*E. antiquorum*	Not specified	N/A	[77]
		*E. chrysocoma*	Not specified	N/A	[78]
	*Excoecaria*	*E. agallocha*	Not specified	N/A	[24]
	*Sebastiana*	*S. adenophora*	Leaves	1.6–13.0 mg/Kg dry weight	[25]
	*Homonoia*	*H. riparia*	Roots	N/A	[43]
	*Macaranga*	*M. triloba*	Leaves	0.19 mg/g dry weight	[79]
	*Alchorneae*	*A. latifolia*	Leaves	0.0007% ^3^	[80]
Fabaceae	*Prosopsis*	*P. juliflora*	Seed oils	N/A	[67]
	*Clitoria*	*C. ternatea*	Roots	12.4 mg/g dry weight	[9]
	*Erythrophleum*	*E. fordii*	Leaves	3.01 mg/Kg dry weight	[24]
Icacinaceae	*Pyrenacantha*	*P. staudtii*	Leaves	N/A	[26]
Lamiaceae	*Clerodendrum*	*C. trichotomum*	Leaves	N/A	[27]
	*Vitex*	*V. trifolia*	Not specified	N/A	[81]
	*Clerodendrum*	*C. bungei*	Not specified	N/A	[82]
Lecythidaceae	*Barringtonia*	*B. racemosa*	Bark of the stems	N/A	[83]
Malvaceae	*Pavonia*	*P. multiflora*	Not specified	N/A	[84]
	*Abroma*	*A. augusta* L.	Leaf	28.80 mg/Kg dry weight	[28]
	*Heritiera*	*H. littoralis*	Leaf	N/A	[85]
	*Bombax*	*B. ceiba* (II)	Leaf	N/A	[29]
	*Microcos*	*M. tomentosa*	Roots	10.08 mg/Kg dry weight	[49]
	*helmiopsis*	*H. sphaerrocarpa*	Leaves	6.56 mg/Kg dry weight	[30]
	*Sterculia*	*S. foetida*	Leaves	0.11 mg/g dry weight	[31]
	*Pterospermum*	*P. heterophyllum*	Roots	12.88 mg/Kg dry weight	[50]
Moraceae	*Ficus*	*F. thonningii* Blume	Roots	0.04 mg/g dry weight	[51]
		*F. aurantiaca*	Stem	N/A	[57]
		*F. foveolata*	Stem	2.9 mg/Kg dry weight	[58]
Myricaceae	*Myrica*	*M. rubra*	Bark	141.00 mg/Kg dry weight	[52]
		*M. cerifera*	Root	N/A	[52]
Myrsinaceae	*Embelia*	*E. schimperi*	Leaves	35 mg/Kg dry weight	[32]
Myrtaceae	*Eugenia*	*E. umbelliflora*	Leaves	N/A	[33]
Ranunculaceae	*Naravelia*	*N. Zeylanica*	Leaves	N/A	[34]
Rhamnaceae	*Ventilago*	*V. leiocarpa*	Stems	N/A	[59]
	*Sageretia*	*S. theezans*	Not specified	N/A	[86]
Rhizophoraceae	*Rhizophora*	*R. stylosa*	Leaves	N/A	[35]
		*R. mangle*	Leaves and stems	0.77 mg/g dry weight	[36]
Rubiaceae	*Mitragyna*	*M. rotundifolia*	Bark	N/A	[87]
Rutaceae	*Vepris*	*V. punctata*	Wood	2.2 mg ^2^	[88]
Sapindaceae	*Cupania*	*C. cinerea*	Bark	0.08 mg/g dry weight	[89]
Sapotaceae	*Mimusops*	*M. elengi*	Seed oils	N/A	[45]
		*M. hexandra*	Bark	14.14 mg/Kg dry weight	[90]
Solanaceae	*Solanum*	*S. macrocarpon*	Cuticular waxes of the leaves	3.5–7.4 ng cm^−2^ *	[91]
Styracaceae	*Styrax*	*S. japonica*	Stem-bark	28.08 mg/Kg dry weight	[92]
Vitaceae	*Vitis*	*V. vinifera*	Leaf	N/A	[37]
	*Tetrastigma*	*T. hemsleyanum*	Not specified	N/A	[93]

^1^ N/A: The authors did not fully provide the taraxerol accumulation information in their findings. ^2^ The amount of starting material for extraction was not stated by the author. ^3^ Taraxerol accumulation data was based on GC-MS analysis without comparison with authentic standard. * The standard was based on the composition of free triterpene and sterol fractions of *S. macrocarpon* leaf cuticular waxes (ng cm^−2^) of leaf surface.
